# Adaptive Fiber-Ring Lasers Based on Isopropanol Filled Microfiber Coupler for High-Sensitivity Temperature Sensing

**DOI:** 10.3390/mi13101697

**Published:** 2022-10-09

**Authors:** Weihao Lin, Jie Hu, Fang Zhao, Siming Sun, Yuhui Liu, Shuaiqi Liu, Feihong Yu, Peng-Un Mak, Sio-Hang Pun, Perry-Ping Shum, Mang-I Vai, Liyang Shao

**Affiliations:** 1 State Key Laboratory of Analog and Mixed-Signal VLSI, University of Macau, Macau 999078, China; 2 Department of Electrical and Electronic Engineering, Southern University of Science and Technology, Shenzhen 518055, China; 3 Department of Electrical and Computer Engineering, University of Macau, Macau 999078, China; 4 Peng Cheng Laboratory, Shenzhen 518005, China

**Keywords:** temperature measurement, fiber ring laser, micro-fiber coupler

## Abstract

We demonstrated a new method for temperature measurement inside a fiber ring laser (FRL) cavity. Different from traditional FRL temperature sensing system which need additional filter working as a sensor, a micro-fiber coupler (MFC) was designed as a beam splitter, filter, and temperature sensor. In addition, isopropanol, a liquid with very high photothermal coefficient, is selectively filled in the MFC in order to improve the sensitivity of the system on temperature. In the dynamic range of 20–40 °C, we obtained a good temperature sensitivity of −1.29 nm/°C, with linear fitting up to 0.998. Benefiting from the advantages of laser sensing, the acquired laser has a 3 – dB bandwidth of less than 0.2 nm and a signal-to-noise ratio (SNR) of up to 40 dB. The proposed sensor has a low cost and high sensitivity, which is expected to be used in biomedical health detection, real-time monitoring of ocean temperature, and other application scenarios.

## 1. Introduction

Optical fiber sensors have been widely recognized after decades of development [1,2,3,4,5]. Differently from traditional electronic sensors, optical fiber has its own indispensable advantages, including but not limited to: electrical insulation, corrosion resistance, variable shape, small size, wide range of measuring objects, and easy reuse [6,7,8,9,10]. Among them, temperature, as the most important parameter for perceiving the external environment, has received the most extensive attention from scientists, and has been widely researched [11,12,13]. Various optical fiber structures have been designed to improve the sensitivity, stability, and practicability of temperature sensing [14,15,16]. Common structures include Mach Zehnder Interferometer (MZI), Fabry Perot Interferometer (FPI), Sagnac Ring, and Fiber Bragg Grating (FBG) [17,18,19,20]. However, due to the characteristics of broadband light sources, there are some unavoidable defects, such as low signal-to-noise ratio and large linewidth, which will cause measurement errors.

In recent years, MFC has been noticed. It has been found that after two ordinary single-mode optical fibers are fused and pulled to one MFC, interference will occur. High sensitivity temperature monitoring can be achieved by filling liquid with a high photothermal coefficient. Wang et al. immersed micro/nano optical fibers in seawater for a dual parameter measurement of salinity and temperature [21]. In order to maintain a stable structure in liquid, they proposed a method of ethyl cellulose ethanol film coating. By coating the coupling region with ethyl cellulose ethanol solvent, it can operate stably in seawater. The temperature sensitivity is −1.13 nm/°C. However, the signal intensity of this structure is below 70 dB, leading to questions about the practicability of monitoring in the real marine environment. Zhao’s and Jian’s group proposed an ultra-high sensitivity optical fiber temperature sensing system based on alcohol filling almost at the same time [22,23]. The temperature sensitivity up to 5 nm/°C was achieved through biconical fiber taper coupling, capillary encapsulation, and isopropanol filling. However, large bandwidth may cause potential measurement errors, and it is still possible to continue improving.

In the past decade, a sensing system based on fiber ring laser cavity has been gradually attracting attention. It is independent of a broadband light source, and has advantages including narrow linewidth, high signal-to-noise ratio, and no burr. Fukushima et al. proposed a dual-pass cascaded-chirped long-period fiber grating as a reflective sensor device, and a wavelength selection element with comb spectrum to achieve a temperature sensor based on erbium-doped fiber laser, achieving a temperature sensitivity of 0.2 nm/°C [24]. However, the cascade structure is complex, not only increasing the system cost but also reducing the stability at the same time. In addition, Lin et al. discovered the vernier effect in the fiber laser cavity by cascading Sagnac rings, which increased the temperature sensitivity by more than four times. However, the complex structure design reduced the signal-to-noise ratio of the system [25]. In the past two years, researchers have gradually realized that the MFC formed by melting two single-mode optical fibers into a taper can replace the coupler of the traditional process to operate in the FRL system. All work is focused on achieving ultra-fast lasers by coating MFC with saturable absorption [26,27,28,29]. As far as we know, the FRL system based on MFC for sensor systems has not been reported yet. 

In this letter, differently from the traditional FRL temperature sensing system, we combined the advantage of MCF and the laser cavity. The MFC works as a sensing element, filter, and coupler. In addition, the isopropanol with ultra-high photothermal coefficient is selectively filled into MFC in order to improve the temperature sensing response characteristics. The manufacturing process and working principle are introduced in detail, and the corresponding temperature characteristics of −1.29 nm/°C in the range of 20 °C–30 °C at the interval of 1 °C are realized. The sensitivity of −1.2 nm/°C is within the range of 20 °C–40 °C, with an interval of 5 °C. Thanks to the characteristics of laser, the signal-to-noise ratio of the designed sensing system is up to 35 dB, and the 3 – dB bandwidth is better than 0.2 nm. The stability of the structure was tested for 3 h, and the wavelength and intensity shift were better than 0.2 nm and 1 dB, respectively. This is of great potential value for the sensors to be used for life and health temperature monitoring, as well as real-time monitoring of ocean temperature.

## 2. Sensor Setup and Principle

**Figure 1:**
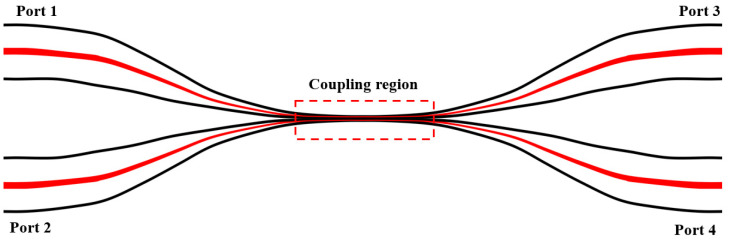
Schematic diagram of tapered MFC.

Figure 1 shows the basic structure of MFC. It consists of three parts: cone, transition, and input and output. When broadband light is emitted to MFC, the lowest order even mode and odd mode are excited simultaneously. Energy is periodically transmitted back and forth between the two modes. The coupling coefficient C between them reciprocates, as shown in formula [22]:

(1) C=3πλ32n1a2·1(1+1V)2

where a is the diameter of MFC and λ is the central wavelength of incident light. V is the normalized frequency, and its expression is [23]:

(2) $$\begin{array}{c} V=\frac{2\pi a}{\lambda }\sqrt{n_{1}^{2}-n_{2}^{2}} \end{array}$$

in which $n_{1}$ and $n_{2}$ is the refractive index of input isopronal and fiber. The actual power in the output side can be expressed as [22]:

(3) $$\begin{array}{c} P=P_{0}sin^{2}\left(CL\right) \end{array}$$

wherein $P_{0}$ represents the total power of the input light and P represents the output power of the light. L is the mutual transmission length of MFC internal modes. The output power depends on the surrounding refractive index, as well as the size and length of the micro nano fiber.

**Figure 2:**
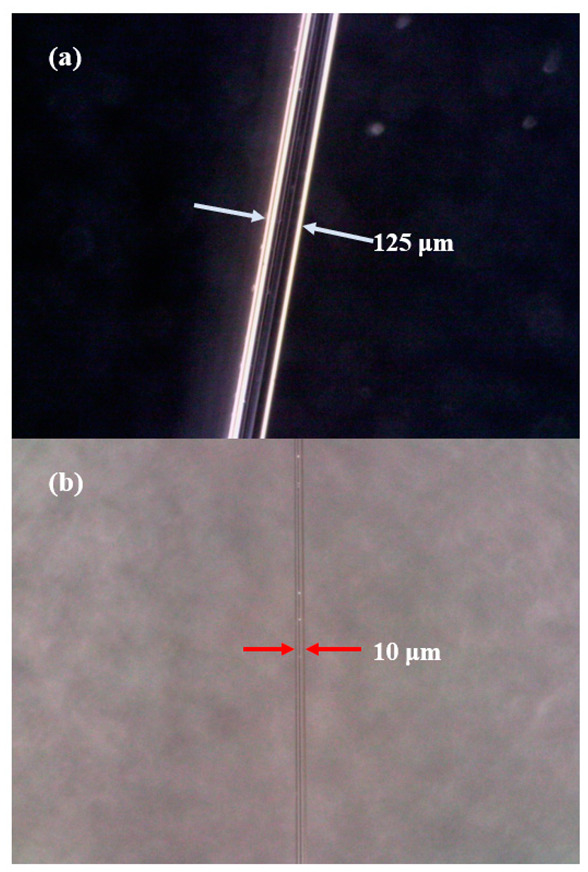
(**a**) Micrograph of single-mode optical fiber; (**b**) micrograph of MFC structure after drawing.

However, the thinner the micro nano fiber is, the greater its loss is. We used an optical fiber taper machine (AFBT-8000, Kepler, Shandong, China) to place two optical fibers in the groove in parallel, and turned on the vacuum pump in order to cause the optical fibers to stick closely to the machine. A common single-mode optical fiber is shown in Figure 2a, and its diameter is 125 microns. By setting the taper distance to 2.3 cm, we have achieved an MFC with a fiber core diameter of 10 microns, two fibers tightly fused and capable of producing interference effects, as shown in Figure 2b. The thinner size is limited by the loss, and cannot generate laser in the experiment. Therefore, the MFC diameter of 10 microns was selected for this experiment.

**Figure 3:**
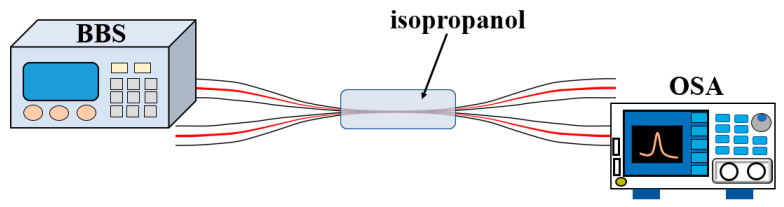
Experimental setup diagram of MCF temperature sensor under broadband light source.

Isopropanol has a very high photothermal response, and its refractive index decreases significantly when the temperature rises. Therefore, effectively filling the liquid can greatly improve the temperature sensitivity. The pre-experiment is shown in Figure 3. Using a broadband light source (Hoyatek ASE-C-N, Shenzhen, China), the isopropanol filled MCF structure was connected, the structure was heated through a thermostat, and, finally, an optical spectrum analyzer (OSA, Yokogawa AQ6370D, Japan) was connected at one of the output ports.

**Figure 4:**
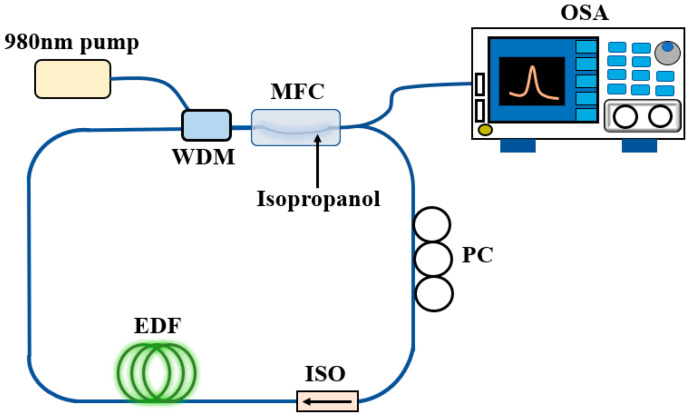
Experimental setup diagram of MCF temperature sensor under fiber ring laser sensing system.

The formal experimental test is shown in Figure 4. The 980 nm pump light source (PL-974-500-FC/APC-P-M, Shenzhen, China) generated the pump light into the annular cavity through the wavelength division multiplexer. The light was amplified by erbium-doped fiber, and the laser near 1550 nm was pumped. The laser interference generated by MFC structure was modulated by temperature. Part of the light was input into OSA, and the polarization controller was used to adjust the polarization state in order to achieve a high power laser output. In addition, the isolator was used to prevent the backscattered light from damaging other devices in the circulator. When the temperature changed, the energy transmitted in the MFC transferred, and the interference peak shift acted as a tunable filter to output lasers of different wavelengths.

## 3. Experimental Results 

**Figure 5:**
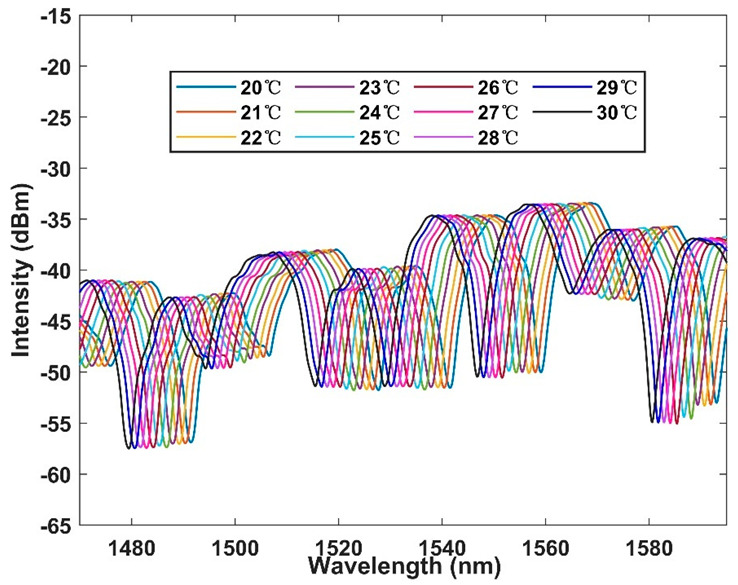
Output spectrum of temperature response with broadband light source (BBS) in the range of 20–30 °C within 1 °C per interval.

**Figure 6:**
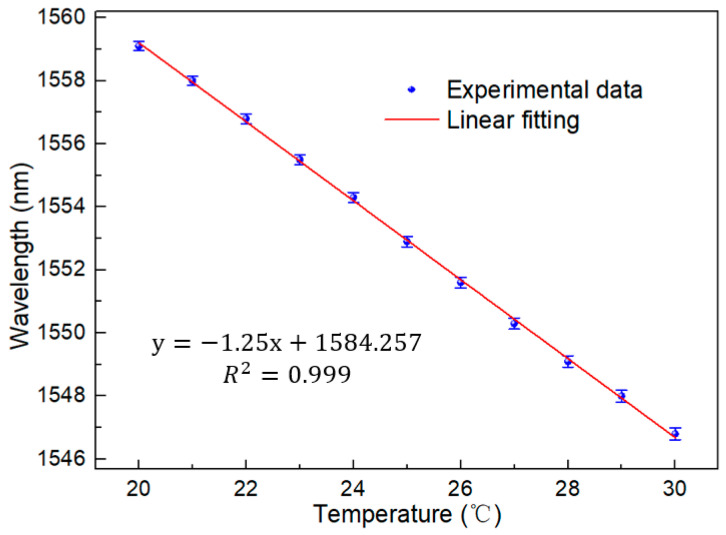
Linear fitting under BBS (20–30 °C).

First, in order to test the performance of the interferometer, the broadband light source was used to test the interferometer in the range of 20–30 °C. The broadband light source entered from one port of the coupler, and one of the output terminals was connected to the OSA. The interval between each temperature is 1 °C, and the test interval is 20 min long, to ensure good thermal conductivity. The test results are shown in Figure 5. It is obvious that with the increase of temperature, there is a blue shift to a short wavelength on the spectrum. We intend to further analyze the sensitivity and linear relationship of the sensor. The linear fitting of spectra at different temperatures is shown in Figure 6. According to the figure, the detection sensitivity is up to −1.29 nm/°C, which is an order of magnitude higher than the sensitivity of an ordinary optical fiber temperature sensor. Moreover, the fitting coefficient is up to 0.999, showing excellent linearity, which proves the feasibility of working in this temperature range.

**Figure 7:**
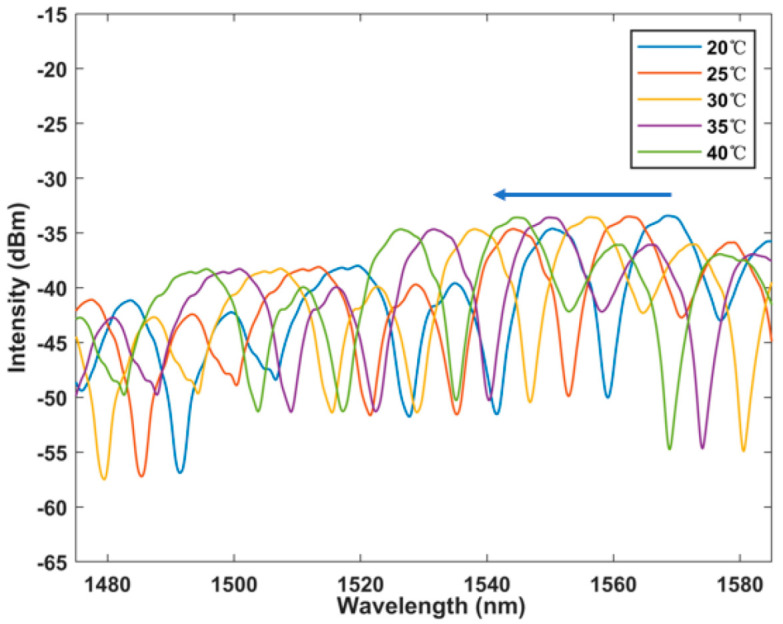
Output spectrum of temperature response with BBS in the range of 20–40 °C within 5 °C per interval.

**Figure 8:**
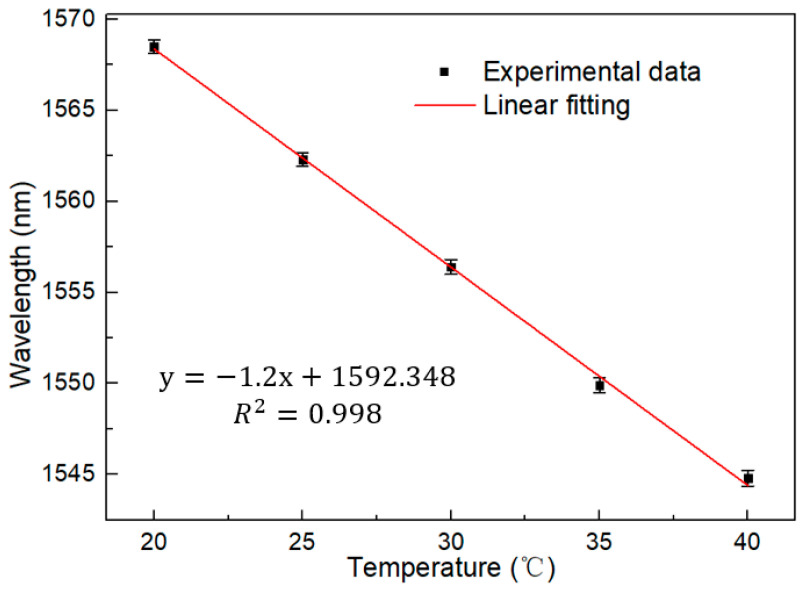
Linear fitting curve under BBS (20–40 °C).

The dynamic range of the designed sensing system was further measured, and the temperature measurement range was expanded from 20 °C to 40 °C at 5 °C intervals. The spectrum picture shown in Figure 7 was obtained, and it can be observed that there was a gradual overlap between the spectra with the increase of temperature. Therefore, the temperature response range could not be further expanded. The effective measurement range is 20 °C to 40 °C. The sensitivity remains at −1.2 nm/°C. The linear fitting curve is shown in Figure 8, and the R square is equal to 0.998. This proves that the feasibility of the sensor as a temperature detection instrument provides a basis for the next step of laser detection.

**Figure 9:**
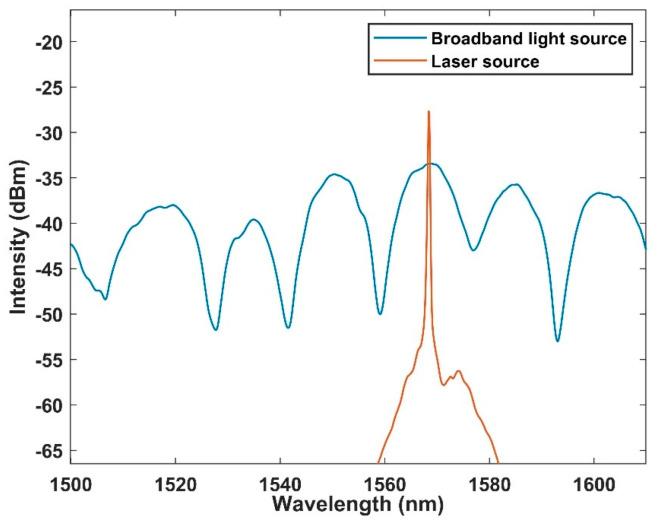
Fitting diagram of interference spectrum and laser spectrum.

**Figure 10:**
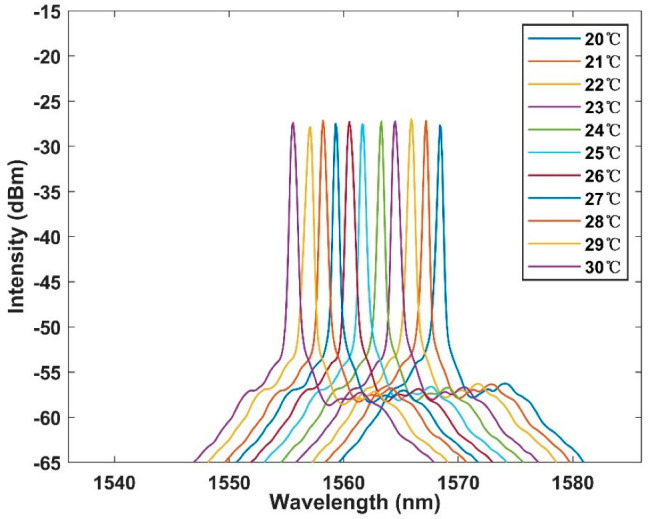
Different output spectra of the laser, varying with temperature (20–30 °C; interval is 1 °C).

**Figure 11:**
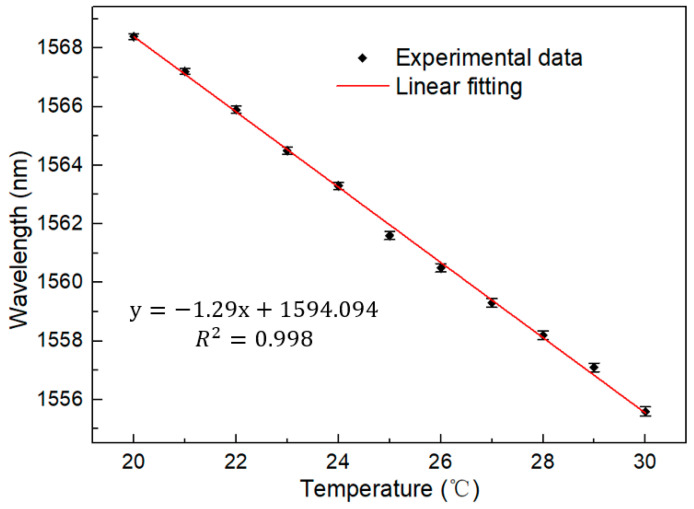
Linear fitting curve of laser changes with temperature (20–30 °C; interval is 1 °C).

Then, we first coupled the interference spectrum and laser spectrum at 20 °C, as shown in Figure 9. It can be found that the peak value of the interference spectrum corresponds to the spectrum of the laser output. It has been proven that MFC works well as a filter and as a coupler. At the same time, it can be observed that the linewidth is less than 0.2 nm and the signal-to-noise ratio is as high as 35 dB. Therefore, we formally monitored the temperature through the FRL system. The test results are shown in Figure 10. It can be found that the wavelength is still blue shifted. The linear fitting curve is shown in Figure 11, and the sensitivity is up to −1.29 nm/°C, which is consistent with broadband light sources. In addition, the R square is 0.998, demonstrating the reliability of the sensing system.

**Figure 12:**
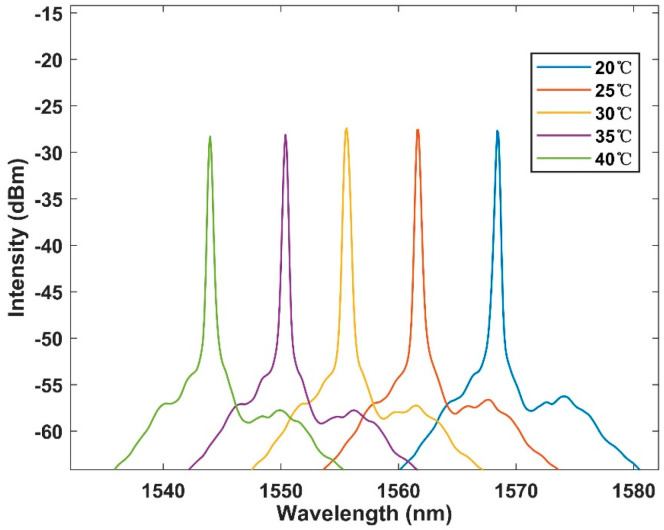
Different output spectra of the laser, varying with temperature (20–40 °C; interval is 5 °C).

**Figure 13:**
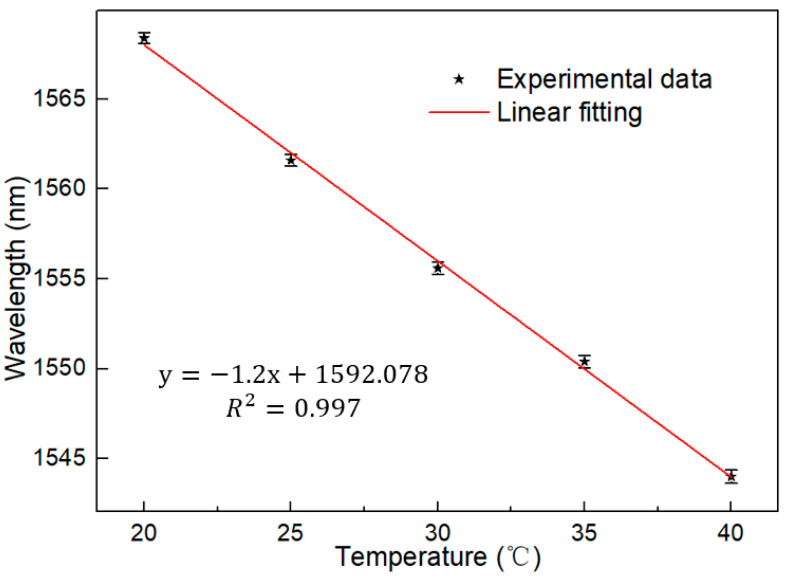
Linear fitting curve of laser changes with temperature (20–40 °C; interval is 5 °C).

Similarly, we measured the temperature in a larger dynamic range, from 20 °C to 40 °C at 5 °C intervals. The temperature corresponding curve and the linear fitting curve are shown in Figure 12 and Figure 13. The sensitivity is up to −1.2 nm/°C. At the same time, the linear fitting degree is kept at 0.997, realizing the wide range of ultra-high sensitivity temperature response. The sensitivity obtained in the experiment is identical to that of the broadband light source, but it retains the characteristics of the laser, namely, narrow linewidth and high signal-to-noise ratio.

**Figure 14:**
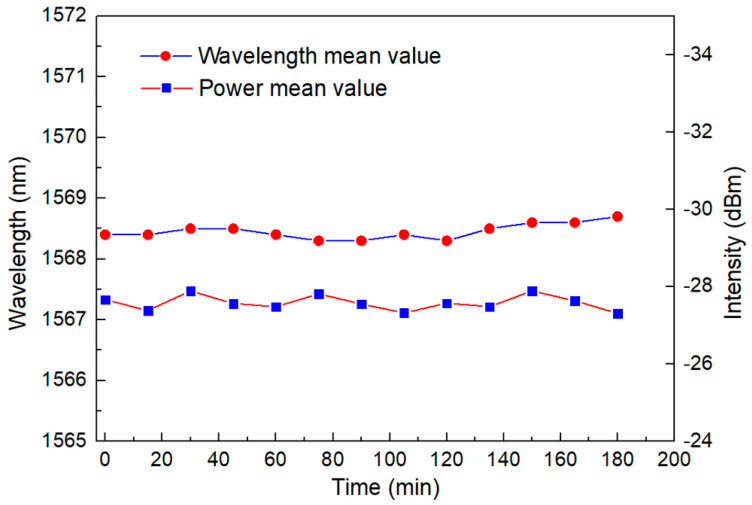
Amplitude diagram of the wavelength and intensity shift within 3 h at 20 °C.

When evaluating the quality of a sensor, its stability should be considered in addition to its sensitivity. This experiment also measured the stability of the laser structure, as shown in Figure 14. Within 3 h, the wavelength shift range was less than 0.2 nm, and the intensity change was better than 1 dB. Table 1 also compares the sensitivity of the temperature sensors of different structures. It can be found that the designed temperature sensor system is more than an order of magnitude higher than the traditional structure.

**Figure 15:**
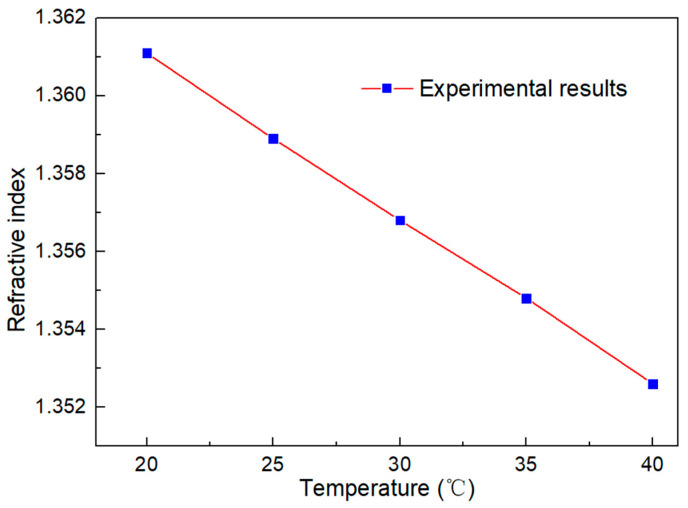
Temperature dependence curve of refractive index of isopropanol.

In order to verify the temperature response characteristics of isopropanol, we tested the change in the isopropanol refractive index at different temperatures through the refractometer, as shown in Figure 15.

## 4. Conclusions

In this study, we proposed a new method for ultra-high temperature measurement. This is the first time that an MFC has been reported to work as a coupler, filter, and sensing system, differently from the traditional FRL temperature sensing system. This new method can reduce cost and improve stability. Thanks to the high photothermal coefficient of isopropanol, the temperature sensitivity is −1.29 nm/°C at a range of 20 °C–30 °C, and −1.2 nm/°C at a range of 20 °C–40 °C. Moreover, benefiting from the FRL system itself, the system has high SNR (−35 dB) and narrow 3 – dB bandwidth (−0.2 nm). This kind of temperature sensor can be used in biomedical engineering and marine temperature detection.

## Figures and Tables

**Table 1:** The sensitivity of temperature sensors of different structures.

Structures	Sensitivity (nm/°C)	Refs.
Peanut-shaped MZI	0.049	[30]
Double spherical cascade	0.306	[31]
Gold-filled PCF	1.3	[32]
Sagnac loop	1.59	[33]
MFC	1.29	This work

## Data Availability

Not applicable.
